# Evaluation of potential antiplatelet effects of CSL112 (Apolipoprotein A-I [Human]) in patients with atherosclerosis: results from a phase 2a study

**DOI:** 10.1007/s11239-018-1644-z

**Published:** 2018-03-26

**Authors:** Paul A. Gurbel, Udaya S. Tantry, Denise D’Andrea, Thomas Chung, John H. Alexander, Kevin P. Bliden, Samuel D. Wright, Pierluigi Tricoci

**Affiliations:** 1 Inova Center for Thrombosis Research and Drug Development, Inova Heart and Vascular Institute, 3300 Gallows Rd, Falls Church, VA 22042 USA; 2 CSL Behring, King of Prussia, PA USA; 3 Duke Clinical Research Institute, Durham, NC USA

**Keywords:** Atherosclerosis, Dual antiplatelet therapy, High-density lipoprotein, Clinical trials, Platelets

## Abstract

CSL112 (Apolipoprotein A-I [Human]), an infusible, plasma-derived apolipoprotein A-I, is being developed to reduce cardiovascular events following acute myocardial infarction (AMI). A predecessor compound (CSL111) demonstrated a potential antiplatelet effect. A phase 2a multicentre, randomised, single-ascending dose study in patients with stable atherosclerotic disease receiving dual antiplatelet therapy (DAPT) assessed the potential additive effects of CSL112 administration on platelet function and increase bleeding risk in the subacute period after AMI. Patients (n = 44) on aspirin (75–325 mg/day) and either clopidogrel (75 mg/day, n = 37) or prasugrel (10 mg/day, n = 7) for > 30 days alongside standard-of-care therapy were randomised to a single dose of placebo or CSL112: 1.7, 3.4, or 6.8 g. Light transmission aggregometry was used to assess platelet aggregation in response to 2 mM arachidonic acid, 5 and 20 µM adenosine diphosphate, and 4 µg/mL collagen, pre-dose (baseline) and up to 48 h post-dosing. Compared to placebo, CSL112 had no clinically meaningful time- or dose-dependent effects on maximum platelet aggregation in response to any agonist, by either dose or renal function subgroup (p > 0.05). Coagulation parameters showed little variation over time or between treatment groups (p > 0.05). CSL112, when co-administered with standard DAPT, did not significantly influence platelet aggregation in response to agonists and is, therefore, not expected to significantly increase bleeding risk when administered with antiplatelet therapies.

**Electronic supplementary material:**

The online version of this article (10.1007/s11239-018-1644-z) contains supplementary material, which is available to authorized users.

## Introduction

The role of statin and antithrombotic therapies in reducing the risk of adverse clinical events in patients with atherosclerotic cardiovascular disease (CVD) is well established [1–3]. Limited additional therapeutic options are available in patients with acute myocardial infarction (AMI) who continue to experience a substantial rate of recurrent ischaemic complications particularly during first month post AMI [4–6]. Novel antithrombotic therapies with potent effects are associated with an increased risk of severe bleeding [7–9]. Infusion of apolipoprotein A-I (apoA-I), in the form of reconstituted high-density lipoprotein (HDL), may facilitate plaque stabilisation and reduce inflammation in patients with vascular disease in the early period after an ischaemic event. CSL112 (Apolipoprotein A-I [Human]) is a novel formulation of apoA-I purified from human plasma and formulated with phosphatidylcholine to yield reconstituted HDL particles [10–12]. CSL112 infusion has shown dramatic elevation of cholesterol efflux capacity (CEC) in healthy subjects [13] and in patients with stable atherosclerotic disease [14] or a recent AMI [15]. CEC measured ex vivo at baseline in a population-based cohort free from CVD was shown to be independently associated with incident atherosclerotic CVD [16, 17]. CSL112 is currently in development to reduce the risk of early recurrent cardiovascular events following AMI with the hypothesis that by enhancing CEC it may lead to plaque stabilisation and reduced events.

The safety, pharmacokinetic (PK) and pharmacodynamic (PD) profiles of CSL112 were assessed in a phase 2a multicentre, randomised, placebo-controlled, single ascending dose (SAD) study (NCT01499420) in patients with stable atherosclerotic disease on DAPT [14]. Prior work on an early formulation of apoA-I, CSL111, revealed an effect on platelet aggregation [18, 19]. We sought to assess whether CSL112 has any antiplatelet effect in patients with stable atherosclerotic CVD treated with antiplatelet medications [dual antiplatelet therapy (DAPT)] and if co-administration may potentially increase bleeding risk.

## Materials and methods

### Study design and patients

This adaptive, multicentre, randomised, parallel-group, double-blind, placebo-controlled, SAD study. Detailed study design, inclusion and exclusion criteria are described elsewhere [14].

Briefly, the study included patients (18–80 years of age) with a documented history of atherosclerotic coronary artery disease/surgical revascularisation or peripheral vascular disease. There was a minimum 1 month time interval between any acute event, revascularisation procedure, or hospitalisation for chest pain, and a patient’s randomisation. Patients were taking DAPT [aspirin (75–325 mg/day) and either clopidogrel (75 mg/day) or prasugrel (10 mg/day)] for a minimum of 30 days before randomisation. All other medications influencing platelet function (e.g., nonsteroidal anti-inflammatory drugs) or coagulation were prohibited from 7 days prior to randomisation.

Patients were screened 3–50 days before randomisation. Patients were asked to fast overnight for 8 h before and after study-drug administration. On Day 1, patients received a 2-h infusion of allocated study drug and remained in the study unit for approximately 48 h after study-drug administration, to perform additional safety and laboratory assessments. A clinical follow-up for assessment of adverse events (AEs) was performed at Day 14, completing the active study period. A follow-up was performed 90 days after study drug administration for a standard virus panel and to assess AEs (Supplementary Fig. 1).

### Study drug administration

Lyophilised CSL112 was reconstituted with sterile water for injection and was dosed based on total protein content. The placebo solution was 0.9% saline. Patients within a cohort were randomised to receive either CSL112 (1.7, 3.4 and 6.8 g) or placebo in a 3:1 ratio administered by intravenous (IV) infusion over 2 h. Randomisation was stratified by renal function: normal renal function (creatinine clearance [CrCl] ≥ 90 mL/min) or mild renal insufficiency (CrCl ≥ 60 to < 90 mL/min), with at least 50% of patients in each dose group having mild renal insufficiency. The study design was adaptive to maximise safety; dose escalation was informed and may have been adjusted based on safety and PK data that emerged during the study. Patients were administered aspirin and either clopidogrel or prasugrel in the morning 2 h prior to the start of the study drug infusion, and at 24-h intervals during the in-house study period.

### Haematology and coagulation parameters

Routine haematology parameters were assessed at baseline and at 8, 12, 24, 36 and 48 h post dose, whereas coagulation parameters were determined at baseline and 24 and 48 h post dose using standard laboratory techniques.

### Light transmittance aggregometry

All centres used the same aggregometer and standardised protocol, and were trained to ensure uniform platelet function measurement techniques. Platelet aggregation was measured at baseline and at 8, 12, 24 and 48 h after administration of the study drug. Blood samples were collected from a peripheral vein into vacutainer tubes (Becton-Dickinson, Franklin Lakes, NJ) containing 3.2% trisodium citrate. Platelet aggregation, induced by 2 mM arachidonic acid (AA), 5 and 20 µM adenosine diphosphate (ADP), and 4 µg/mL collagen, was measured in platelet-rich plasma using the Chrono-log Optical Aggregometer, Model 490-4DR with internal AGGRO/LINK® Interface (Chrono-log corporation, Havertown, PA) as described previously [20]. The final extent of aggregation, measured at 6 min after agonist addition, and the maximal extent of aggregation were expressed as the percent change in light transmittance from baseline, with platelet-poor plasma as a reference.

### Study endpoints

The endpoint for this sub-study is the absolute change from baseline in platelet aggregation as assessed by dose group and by subgroups defined by renal function.

### Sample size

This study was not designed to test specific hypotheses so the safety analysis was descriptive and no formal sample-size calculation was conducted. The sample size was chosen so that relatively common safety events would have a high likelihood of being observed during the 14 days after infusion; events occurring with 1, 2 and 10% rate would have a 36, 59 and 99% chance of being observed, respectively.

### Statistical analysis

The platelet function analyses were pre-specified as exploratory endpoints. Statistical significance (p < 0.05) for change from baseline in maximal aggregation among the treatment groups at any time point was assessed by ANOVA and was performed by the Duke Clinical Research Institute using SAS version 9.2. Summary statistics for the change from baseline for the four platelet function parameters, measured by light transmission aggregometry, were presented as the mean and 95% confidence interval (CI) for the mean. The Wilcoxon signed-rank test was used to assess statistical significance within groups for change from baseline. All other results were presented as the mean ± standard deviation (SD) including the baseline comparison of platelet function parameters, haematology, and coagulation parameters. SAS version 9.4 was used for these analyses, which were not adjusted for multiplicity.

## Results

### Patient allocation and baseline characteristics

Of 45 patients randomised, one patient in the 6.8 g CSL112 group withdrew from the study before receiving study drug and was not considered in further analyses. The baseline characteristics were described previously [14]. The majority of randomised patients were male (73%), and Caucasian (79%).

There was no indication of a major imbalance of prevalent diseases or concurrent medications among the treatment groups. Patients had coronary artery disease or peripheral artery disease with a high prevalence of cardiovascular risk factors. All patients were on DAPT during the active treatment period and most patients were treated with statins.

#### Platelet aggregation

Since all patients were receiving DAPT prior to administration of CSL112, AA-, collagen- and ADP-induced platelet aggregation levels were low (both maximum and final extent) and there were no clinically meaningful differences between groups at baseline (Table 1 and Supplementary Table 1).

**Table 1:** Baseline maximum extent platelet aggregation according to CSL112 treatment groups

Agonist	Placebo (n = 11)	CSL112		
		1.7 g (n = 7)	3.4 g (n = 12)	6.8 g (n = 14)
2 mM AA	5.2 ± 4.6	8.2 ± 11.8	8.7 ± 5.7	13.4 ± 9.6^a^
5 µM ADP	19.4 ± 18.0	23.8 ± 16.7	13.5 ± 12.8	19.2 ± 13.9
20 µM ADP	30.9 ± 23.7	31.8 ± 19.5	27.7 ± 17.0	25.6 ± 16.3
4 µg/mL collagen	23.9 ± 25.1	26.7 ± 18.2	33.0 ± 31.4	20.5 ± 17.2

Values shown are mean ± SD. Each individual dose comparison with placebo was based on Wilcoxon rank-sum test. Individual comparisons with placebo were unadjusted for multiple comparisons. p > 0.05 for all dose groups and agonists except 2 mM arachidonic acid for 6.8 g dose group. ^a^p = 0.0145

*AA* arachidonic acid, *ADP* adenosine diphosphate

**Fig. 1:**
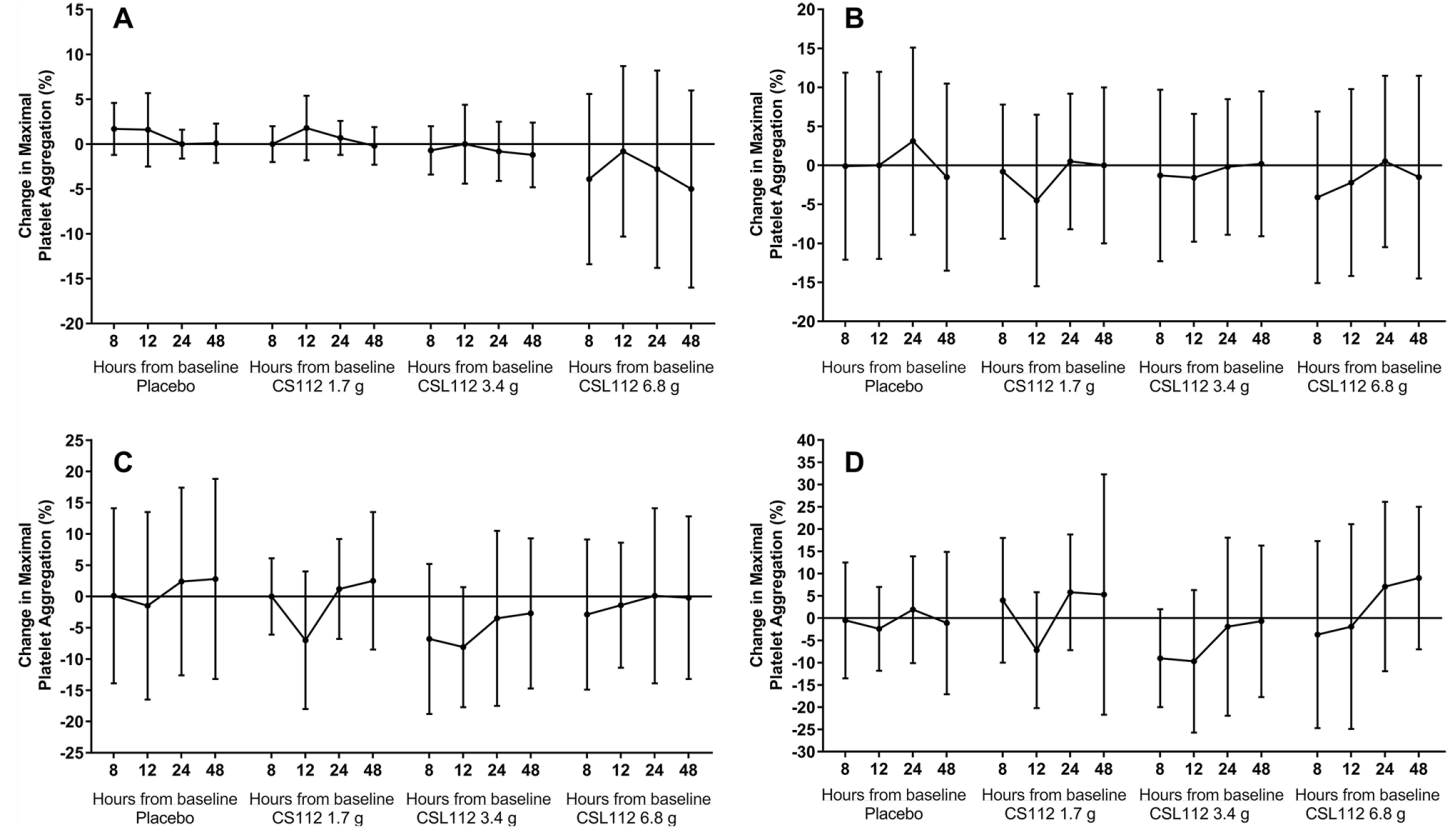
Change in maximal platelet aggregation in the post-dosing period. Change in maximal aggregation (mean ± SD) was assessed by ANOVA (p > 0.05 for all tests) in the post-dosing period induced by 2 mM arachidonic acid (**a**), 5 μM adenosine diphosphate (**b**), 20 μM adenosine diphosphate (**c**) and 4 μg/mL collagen (**d**)

With regard to the change in platelet function parameters after administration of CSL112, no time- or dose-dependent effects on maximal platelet aggregation in response to any agonist (p > 0.05 for all tests) compared to placebo (Fig. 1) were observed. Indeed, with only one exception, there were no clinically meaningful within group changes in the platelet aggregation parameters for either placebo or the combined CSL112 group overall or within renal function groups (Table 2) at the end of the observation period (48 h).

**Table 2:** Change in maximum extent platelet aggregation according to renal function groups

Mean (95% CI)	Normal renal function		Mildly impaired renal function		Overall	
	Placebo (n = 5)	CSL112 (n = 14)	Placebo (n = 6)	CSL112 (n = 15)	Placebo (n = 11)	CSL112 (n = 32)
2 mM AA						
Baseline	3.8 (− 0.1, 7.7)	10.2 (5.1, 15.3)	6.3 (0.4, 12.2)	9.4 (5.2, 13.5)	5.2 (2.1, 8.3)	10.6 (7.4, 13.9)
Change at 8 h	0.6 (− 1.5, 2.7)	−3.8 (− 9.7, 2.0)	0.8 (− 3.6, 5.3)	−3.6 (− 6.6,−0.5)^a^	0.7 (− 1.4, 2.9)	−4.0 (− 6.8, − 1.2)^b^
Change at 12 h	2.8 (− 4.3, 9.9)	−3.2 (− 7.8, 1.4)	−2.2 (− 8.4, 4.0)	−1.0 (− 3.4, 1.4)	0.3 (− 3.8, 4.4)	−2.5 (− 4.9, 0.0)
Change at 24 h	−0.2 (− 1.8, 1.4)	−4.9 (− 10.5, 0.8)	−1.8 (− 6.3, 2.6)	−1.8 (− 4.5, 0.9)	−1.1 (− 3.2, 1.1)	−3.1 (− 5.8, − 0.4)
Change at 48 h	0.4 (− 2.5, 3.3)	−6.3 (− 11.7, − 0.9)^b^	−0.5 (− 2.9, 1.9)	−2.1 (− 4.5, 0.3)	−0.1 (− 1.6, 1.4)	−4.5 (− 7.4, − 1.7)^c^
5 µM ADP						
Baseline	21.8 (− 2.2, 45.8)	21.1 (14.2, 28.0)	17.3 (− 1.9, 36.6)	14.6 (6.0, 23.3)	19.4 (7.3, 31.4)	18.3 (13.1, 23.4)
Change at 8 h	1.2 (− 11.7, 14.1)	−6.6 (− 12.0, − 1.2)^a^	−1.2 (− 15.9, 13.5)	2.3 (− 4.0, 8.6)	−0.1 (− 8.1, 7.9)	−2.1 (− 5.9, 1.8)
Change at 12 h	6 (− 1.6, 13.6)	−5.2 (− 12.2, 1.8)	−14.2 (− 39.4, 11.0)	1 (− 3.5, 5.5)	−4.1 (− 16.7, 8.5)	−2.3 (− 6.1, 1.5)
Change at 24 h	8.2 (− 5.9, 22.3)	−1.4 (− 7.3, 4.4)	0.2 (− 12.0, 12.3)	2.6 (− 2.8, 7.9)	3.8 (− 4.0, 11.7)	0.7 (− 2.9, 4.3)
Change at 48 h	2.6 (− 8.8, 14.0)	−4.7 (− 9.5, 0.1)	−5.0 (− 19.6, 9.6)	5.2 (− 2.4, 12.8)	−1.5 (− 9.7, 6.6)	0.2 (− 4.0, 4.4)
20 µM ADP						
Baseline	34 (2.9, 65.1)	29.1 (19.3, 38.8)	28.3 (2.5, 54.1)	26.5 (17.0, 36.0)	30.9 (15.0, 46.8)	28.3 (22.1, 34.5)
Change at 8 h	1.8 (− 16.0, 19.6)	−6.2 (− 12.4, 0.1)	2.7 (− 12.4, 17.8)	−2.3 (− 10.2, 5.6)	2.3 (− 6.9, 11.4)	−3.7 (− 8.1, 0.6)
Change at 12 h	5 (− 15.0, 25.0)	−6.6 (− 11.9, − 1.3)^a^	−8.6 (− 25.7, 8.5)	−3.4 (− 10.1, 3.2)	−1.8 (− 13.1, 9.5)	−4.9 (− 8.6, − 1.2)^a^
Change at 24 h	6.2 (− 13.7, 26.1)	−3.6 (− 11.8, 4.7)	−1.0 (− 17.5, 15.5)	0.1 (− 6.8, 7.1)	2.3 (− 8.1, 12.7)	−1.5 (− 6.2, 3.2)
Change at 48 h	8.6 (− 6.7, 23.9)	−3.5 (− 10.6, 3.6)	−0.8 (− 20.5, 18.8)	1.6 (− 6.3, 9.5)	3.5 (− 7.4, 14.3)	−0.8 (− 5.5, 3.8)
4 µg/mL collagen						
Baseline	31.8 (1.5, 62.1)	29 (17.1, 40.9)	17.3 (− 9.8, 44.5)	27 (11.8, 42.2)	23.9 (7.0, 40.8)	27.3 (18.7, 35.8)
Change at 8 h	−4.2 (− 26.8, 18.4)	−5.2 (− 17.4, 7.1)	1.8 (− 7.9, 11.6)	−5.7 (− 23.0, 11.6)	−0.9 (− 10.1, 8.2)	−4.6 (− 13.9, 4.6)
Change at 12 h	−3.2 (− 17.7, 11.3)	−11.3 (− 22.8, 0.3)	−4.2 (− 18.4, 10.0)	−0.8 (− 10.4, 8.8)	−3.7 (− 11.5, 4.1)	−5.1 (− 11.8, 1.7)
Change at 24 h	−2.0 (− 18.6, 14.6)	0.7 (− 10.8, 12.2)	4.2 (− 7.5, 15.9)	−0.1 (− 17.3, 17.1)	1.4 (− 6.7, 9.4)	0.8 (− 8.2, 9.7)
Change at 48 h	−4.4 (− 29.7, 20.9)	3.6 (− 5.7, 13.0)	−0.5 (− 17.6, 16.6)	3.3 (− 15.0, 21.5)	−2.3 (− 14.0, 9.4)	2.8 (− 6.1, 11.7)

Within-group p value on change from baseline is based on Wilcoxon signed-rank test.

^a^p < 0.05; ^b^p < 0.01; ^c^p < 0.001

Similarly, there were no clinically meaningful differences in maximal platelet aggregation in response to any agonist between placebo and the combined CSL112 group either overall or any groups defined by renal function (Table 2) when reviewing overlap between the 95% CIs at 48 h.

### Haematology and coagulation parameters

No clinically meaningful differences in haematology parameters were observed at any time point when compared to baseline, and between placebo and the CSL112 groups (Table 3). Similarly, there were no clinically meaningful differences in activated partial prothrombin time and prothrombin international normalised ratio at any time point compared to baseline values and also between placebo and treatment groups based on CSL112 dose or renal function (Table 4 and Supplementary Table 2). Although some individual time points reached statistical significance, the absolute change from baseline was small in magnitude and not thought to be clinically significant. Note, that this is based on nominal p values without any multiplicity adjustment for the large number of comparisons assessed.

**Table 3:** Haematology parameters

	Placebo (n = 11)	CSL112		
		1.7 g (n = 7)	3.4 g (n = 12)	6.8 g (n = 14)
Haematocrit (L/L)				
Baseline	0.424 ± 0.043	0.434 ± 0.058	0.451 ± 0.046	0.429 ± 0.071
6 h	0.412 ± 0.039	0.434 ± 0.065	0.459 ± 0.051	0.438 ± 0.048
12 h	0.399 ± 0.042	0.427 ± 0.069	0.446 ± 0.056	0.427 ± 0.047
24 h	0.406 ± 0.040	0.426 ± 0.064	0.457 ± 0.051	0.419 ± 0.048
36 h	0.404 ± 0.046	0.423 ± 0.068	0.447 ± 0.059	0.426 ± 0.052
48 h	0.404 ± 0.052	0.407 ± 0.062	0.441 ± 0.054	0.409 ± 0.047^a^
Haemoglobin (g/L)				
Baseline	131 ± 13.38	134 ± 21.94	136 ± 19.16	137 ± 24.13
6 h	128 ± 13.42^a^	134 ± 22.05	141 ± 17.60	140 ± 16.48
12 h	125 ± 13.80^a^	129 ± 21.61^a^	136 ± 18.40^a^	136 ± 14.37^a^
24 h	128 ± 14.72	130 ± 23.16	136 ± 20.46	136 ± 17.53
36 h	127 ± 15.66	130 ± 24.10	134 ± 20.61	136 ± 16.91
48 h	127 ± 16.69	125 ± 23.50	132 ± 19.86^a^	132 ± 16.16^a^
Leukocytes (× 10^9^/L)				
Baseline	6.72 ± 1.38	5.93 ± 0.61	6.76 ± 1.24	7.56 ± 1.96
6 h	6.52 ± 1.82	6.10 ± 1.08	6.34 ± 0.96	6.98 ± 1.44
12 h	6.62 ± 1.66	6.50 ± 0.93	6.68 ± 0.95^a^	7.55 ± 1.30
24 h	6.59 ± 1.43	6.71 ± 1.35	6.46 ± 1.31	7.06 ± 1.54^a^
36 h	6.67 ± 1.21	6.49 ± 0.85	6.55 ± 0.90	7.81 ± 1.68
48 h	6.45 ± 1.30	5.72 ± 1.02	6.60 ± 1.41	7.06 ± 1.57
Platelets (× 10^9^/L)				
Baseline	221 ± 51	219 ± 21	192 ± 51	244 ± 52
6 h	203 ± 41^a^	221 ± 30	178 ± 32	248 ± 43
12 h	211 ± 42	207 ± 29	177 ± 35	244 ± 40
24 h	214 ± 65	216 ± 28	195 ± 54	248 ± 42
36 h	212 ± 66	215 ± 29	182 ± 67	250 ± 58
48 h	221 ± 77	216 ± 17	182 ± 47	249 ± 50

Values shown are mean ± SD. Within-group p value on change from baseline is based on Wilcoxon signed-rank test.

^a^p > 0.05 except

**Table 4:** Coagulation parameters according to CSL112 treatment groups

	Placebo n = 11	CSL112		
		1.7 g (n = 7)	3.4 g (n = 12)	6.8 g (n = 14)
aPTT (s)				
Baseline	25.9 ± 0.95	28.1 ± 3.99	28.4 ± 8.97	25.1 ± 1.48
24 h	27.1 ± 2.51	27.1 ± 2.69	26.9 ± 5.69	24.4 ± 2.12
48 h	26.2 ± 3.45	28.4 ± 3.57	26.9 ± 6.41	25.0 ± 1.62
Prothrombin INR				
Baseline	0.95 ± 0.05	0.96 ± 0.06	1.01 ± 0.05	1.01 ± 0.08
24 h	0.98 ± 0.06	1.01 ± 0.07	1.04 ± 0.05	1.02 ± 0.07
48 h	1.07 ± 0.23	1.03 ± 0.10	1.02 ± 0.06	1.01 ± 0.07

Values shown are mean ± SD. Within-group p value on change from baseline is based on Wilcoxon signed-rank test. p > 0.05 at all timepoints for all dose groups

*aPTT* activated partial thromboplastin time, *INR* international normalised ratio

### Adverse events

During the active treatment period, 3/11 (27.3%) patients in the placebo group and 16/33 (48.5%) in the combined CSL112 group experienced at least one study drug-related AE. All AEs were mild in intensity except one moderate intensity serious AE (recurrence of atrial fibrillation) observed in the placebo group. There was no pattern of higher frequency of study drug-related AEs in patients with mild or moderate renal insufficiency compared with those with normal renal function (data not shown). No overt bleeding events occurred during the study. AEs indicative of bruising at the IV administration site or venepuncture site (verbatim term) were reported. Infusion site haematoma and injection site haematoma were reported for three patients. All were grade 1 in intensity, assessed as related to treatment by the investigator and occurred in the 3.4 (1 event) or 6.8 g (2 events) dose groups. Grade 1 vessel puncture site haematoma (four events) was reported for three patients. In two patients, two events were assessed as related to treatment by the investigator, one each in the 1.7 and 6.8 g dose groups.

## Discussion

We have demonstrated that when co-administered with standard DAPT, CSL112 administered at 1.7, 3.4, and 6.8 g doses does not significantly influence platelet aggregation in response to AA, ADP, and collagen. Although the change in AA from baseline reached statistical significance for a within-group change for the combined CSL112 group versus placebo, the difference may be likely attributed to regression to the mean rather than any true treatment difference. Further, there were no clinically significant differences between renal function groups. Finally, there were no clinically meaningful differences in coagulation parameters during CSL112 administration in these stable atherosclerotic disease patients treated with DAPT. Based on these data, it is not anticipated that CSL112 will affect haemostasis when administered with concomitant antiplatelet therapies in the immediate or post-AMI setting.

During the development of CSL112, infusion of a prototype formulation, CSL111, was associated with a reduction in platelet aggregation in response to various agonists in healthy volunteers and in type 2 diabetes mellitus (T2DM) patients [18, 19]. When 13 T2DM patients were infused with placebo or CSL111 (20 mg/kg/hour) for 4 h, there was an ~ 1.4-fold increase in plasma HDL cholesterol levels and > 50% reduction in the ADP-, collagen-, and collagen-related peptide–induced platelet aggregation with CSL111, an effect that persisted in washed platelets. Furthermore, similar inhibitory effects were demonstrated in in vitro studies with washed platelets from healthy individuals [18]. We postulated that CSL112 would not have an effect on platelets or platelet membranes because of changes in its formulation: it is known that depletion of cholesterol from platelet membranes renders them less responsive to agonists [21, 22]. It is further known that cholesterol diffuses out of platelets and into reconstituted HDL and the extent of this diffusion depends on the phospholipid content of the HDL. Because CSL112 is formulated with threefold less phospholipid than CSL111 [12], its ability to deplete platelet cholesterol and compromise platelet function was expected to be threefold reduced. Indeed, we could not demonstrate any significant effect on platelet aggregation upon infusion of CSL112 in healthy volunteers as measured using the PFA-100 system with collagen/ADP- or collagen/epinephrine stimulation [23]. There was no apparent dose response relationship in measurements of platelet function (closure time) at 6, 12, and 24 h after dosing whether measured after an initial infusion or at similar time points after the fourth of four weekly infusions. After the first dose, changes from baseline in mean closure time at 6, 12 and 24 h ranged from − 4.9 to + 14.0 s for placebo and from − 8.1 to + 16.8 s among the CSL112 treatment groups [23].

The current study further supports the hypothesis that the CSL112 formulation has reduced potential for antiplatelet effect as compared with CSL111. Other than mild bruising at the infusion site or venepuncture site, no serious bleeding events were observed. One patient in the 6.8 g dose group with normal renal function was concomitantly treated with a nonsteroidal anti-inflammatory drug and included in the platelet function analysis. This was assessed as having minimal impact on the overall study results.

The current study is limited by the small sample size in each treatment group, particularly with respect to assessment by renal function group. Participation of patients with moderate renal impairment was prohibited by an amendment to the protocol to fulfil a health authority request and as a result the sample size was small in this renal function subgroup (n = 3). Another limitation is the absence of a control group of patients not treated with DAPT. Finally, for ethical reasons the study did not include the ‘positive control’ of CSL111 and, therefore, was not comparative. Rather, the study’s goal was to address effects of CSL112 on platelet function in a defined patient population with atherosclerotic CVD on DAPT prior to proceeding to late stage development.

## Conclusions

CSL112, when co-administered with standard DAPT, does not significantly influence platelet aggregation in response to AA, ADP and collagen. Based on these data, it is not anticipated that CSL112 will affect haemostasis when administered with standard antiplatelet therapies in AMI patients or increase bleeding risk in the subacute period after MI.

## Electronic supplementary material

Below is the link to the electronic supplementary material.

Supplementary material 1 (DOCX 173 KB)
