# Supplementary material for: Evaluation of potential antiplatelet effects of CSL112 (Apolipoprotein A-I [Human]) in patients with atherosclerosis: results from a phase 2a study
Source: J Thromb Thrombolysis. 2018 Mar 26;45(4):469–76. doi: 10.1007/s11239-018-1644-z (PMC5889770; doi:10.1007/s11239-018-1644-z)
Supplement: Supplementary file 1 — Supplementary material 1 (DOCX 173 KB) [file 11239_2018_1644_MOESM1_ESM.docx]

**Evaluation of Potential Antiplatelet Effects of CSL112 (Apolipoprotein A-I [Human]) in Patients with Atherosclerosis: Results from a Phase 2a Study**

Paul A. Gurbel,^1^ Udaya S. Tantry,^1^ Denise D’Andrea,^2^ Thomas Chung,^2^ John H. Alexander,^3^ Kevin P. Bliden,^1^ Samuel D. Wright^2^ and Pierluigi Tricoci^3^

**Supplementary material**

**Supplementary Figure 1.** **Study Flow Diagram**


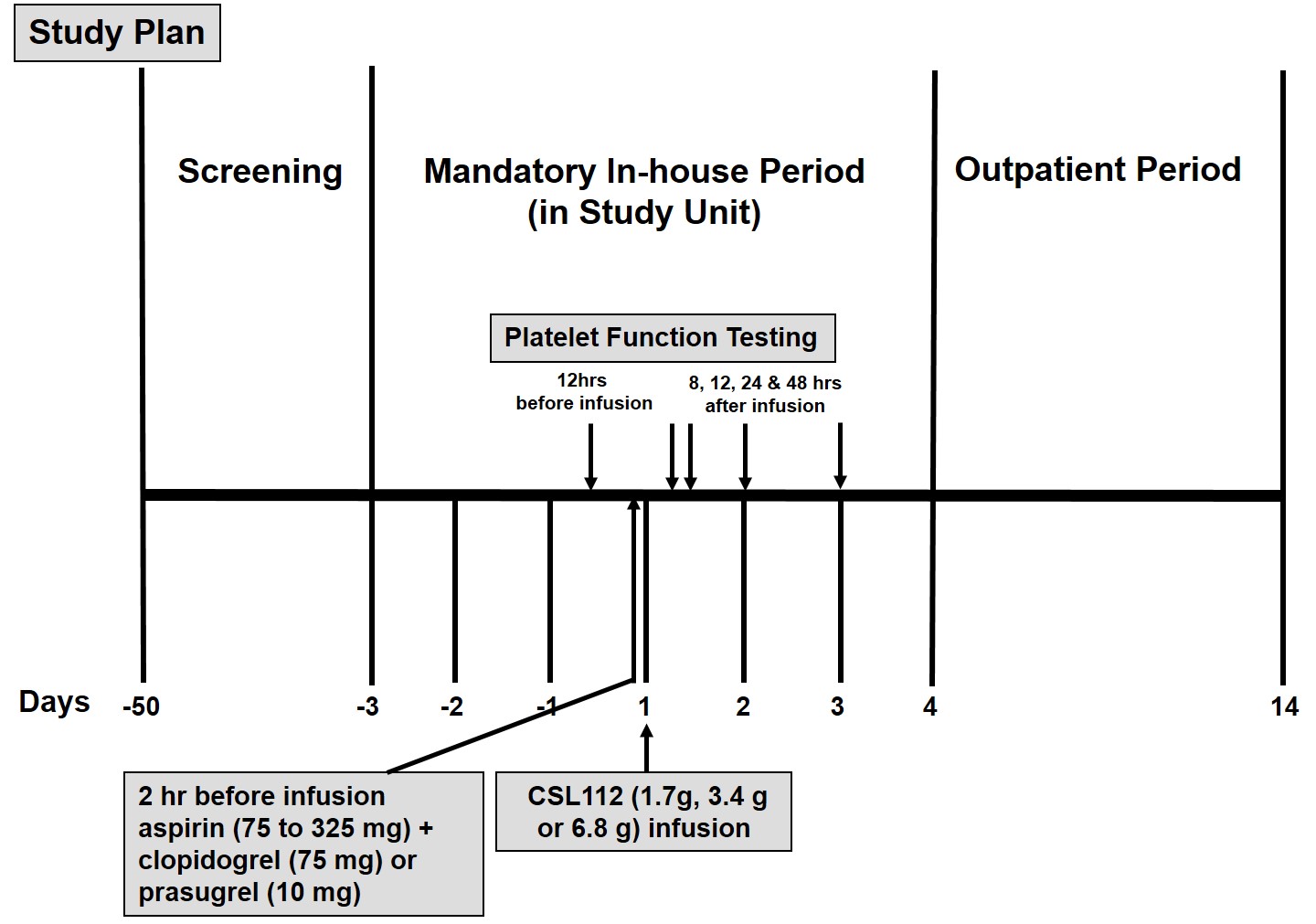


**Supplementary Table 1. Baseline maximum extent platelet aggregation according to renal function groups**

| **Agonist** | **Normal renal function** | | **Mildly impaired renal function** | | **Moderately impaired renal function*^a^*** | |
| --- | --- | --- | --- | --- | --- | --- |
|  | **Placebo**  **(n=5)** | **CSL112**  **(n=14)** | **Placebo**  **(n=6)** | **CSL112**  **(n=15)** | **Placebo**  **(n=0)** | **CSL112**  **(n=3)** |
| **2 mM AA** | 3.8 ± 3.1 | 10.2 ± 9.2 | 6.3 ± 5.6 | 9.4 ± 7.2 | - | 18.7 ± 14.0 |
| **5 μM ADP** | 21.8 ± 19.3 | 20.6 ± 12.3 | 17.3 ± 18.3 | 14.6 ± 15.0 | - | 22.3 ± 19.5 |
| **20 μM ADP** | 34.0 ± 25.1 | 27.5 ± 16.5 | 28.3 ± 24.6 | 26.5 ± 16.4 | - | 33.3 ± 24.7 |
| **4 μg/mL collagen** | 31.8 ± 24.4 | 27.5 ± 20.7 | 17.3 ± 25.9 | 27.0 ± 27.4 | - | 20.3 ± 24.9 |

Values shown are mean ± standard deviation. Comparison between overall CSL112 doses and placebo was based on Wilcoxon rank-sum test. P>0.05 for all dose groups. *^a^*Patients with moderate renal impairment were excluded from participation in the study after an amendment to the protocol and no subjects with moderate renal impairment were randomised to placebo prior to the protocol amendment.

AA, arachidonic acid; ADP, adenosine diphosphate

**Supplementary Table 2. Coagulation parameters according to renal function groups**

|  | **Normal renal function** | | **Mildly impaired renal function** | | **Moderately impaired renal function** | |
| --- | --- | --- | --- | --- | --- | --- |
|  | **Placebo (n=5)** | **CSL112 (n=15)** | **Placebo (n=6)** | **CSL112 (n=15)** | **Placebo (n=0)** | **CSL112 (n=3)** |
| **aPTT (s)** | | | | | | |
| Baseline | 25.5 ± 1.0 | 27.2 ± 8.5 | 26.3 ± 0.8 | 26.9 ± 2.7 | - | 24.6 ± 0.4 |
| 24 hours | 26.2 ± 1.2 | 26.3 ± 5.6 | 27.8 ± 3.0 | 25.6 ± 2.0 *^a^* | - | 24.9 ± 1.2 |
| 48 hours | 25.6 ± 1.7 | 27.4 ± 6.1 | 26.9 ± 4.8 | 25.5 ± 1.8 | - | 25.4 ± 0.9 |
| **Prothrombin INR** | | | | | | |
| Baseline | 0.94 ± 0.06 | 0.97 ± 0.05 | 0.96 ± 0.06 | 1.03 ± 0.07 | - | 1.00 ± 0.00 |
| 24 hours | 0.98 ± 0.08 | 1.03 ± 0.06 *^a^* | 0.98 ± 0.04 | 1.02 ± 0.07 | - | 1.07 ± 0.06 |
| 48 hours | 1.00 ± 0.10 | 1.01 ± 0.06 | 1.14 ± 0.31 | 1.01 ± 0.07 | - | 1.10 ± 0.10 |

Values shown are mean ± standard deviation. Within-group p-value on change from baseline is based on Wilcoxon signed-rank test. P>0.05 except *^a^*. Patients with moderate renal impairment were excluded from participation in the study after an amendment to the protocol and no subjects with moderate renal impairment were randomised to placebo prior to the protocol amendment.

aPTT, activated partial thromboplastin time; INR, international normalised ratio
